# Microplastic impacts archaeal abundance, microbial communities, and their network connectivity in a Sub-Saharan soil environment

**DOI:** 10.1093/femsec/fiaf085

**Published:** 2025-09-02

**Authors:** Stephan Rohrbach, Gerasimos Gkoutselis, Linda Hink, Alfons R Weig, Gerhard Rambold, Marcus A Horn

**Affiliations:** Institute of Microbiology, Leibniz University Hannover, 30419 Hannover, Germany; Department of Mycology, University of Bayreuth, 95440 Bayreuth, Germany; Institute of Microbiology, Leibniz University Hannover, 30419 Hannover, Germany; Genomics and Bioinformatics, University of Bayreuth, 95440 Bayreuth, Germany; Department of Mycology, University of Bayreuth, 95440 Bayreuth, Germany; Institute of Microbiology, Leibniz University Hannover, 30419 Hannover, Germany

**Keywords:** community assembly mechanisms, metabarcoding, microplastics, pathogens, plastisphere, terrestrial ecosystems

## Abstract

Unmanaged plastic waste in Sub-Saharan Africa pollutes large areas and degrades into microplastics (MPs). Surfaces of MP are colonized by bacteria and fungi, resulting in the plastisphere. Plastispheres from high population hotspots on the African continent enrich pathogenic fungi, posing a potential threat to human health. Prokaryotes in such plastispheres are unknown to date. Thus, we analysed the prokaryotic microbiome of native plastisphere and soil by 16S rRNA gene amplicon sequencing, with a focus on community assembly mechanisms and putative pathogenic bacteria. A strong plastic-dependent depletion of archaeal ammonia oxidizing Nitrososphaeraceae was observed. Prokaryotic but not archaeal beta diversity significantly differed between plastisphere and soil microbiomes. The prokaryotic pathogenic potential in the plastisphere was marginally increased relative to soil, suggesting that MP is a driver for fungal rather than bacterial pathogens. Null model comparisons revealed a moderately stronger effect of deterministic selection events in the plastisphere than in soil. We observed a severe disruption of cooccurrence network connectivity in plastisphere communities in contrast to bulk soil communities. This study closes the knowledge gap on plastic debris in Sub-Saharan terrestrial environments, and the observed effects on archaea and cooccurrence networks suggest negative impacts on nitrification and stability of microbial communities.

## Teaser

Sub-Saharan microplastic is ubiquitous, depletes archaea, disrupts microbial community networks impacting community stability and development, as well as impairs genetic potentials for nitrification. Terrestrial microplastic-associated vectoring of bacterial pathogens is negligible.

## Introduction

Indispensable biopolymers in nature inspired humanity to mimic their function leading to the invention and mass-production of synthetic polymers, commonly called plastics, with a today well-known high variety (Geyer et al. 2017). Plastics outperform biopolymers in terms of durability, making it useful materials in construction and other areas of life. These polymers have reduced, though not omitted natural resource exploitation and environmental burdens associated with glass, aluminum, or cotton production at the expense of fossil fuels (Ram 1997, Andrady and Neal 2009). Despite these partially positive effects, plastic has become a globally relevant environmental pollutant (Persson et al. 2022). Production increase, misdirected disposal, and lack of sufficient waste- and recycling-management led to accumulation of plastics in natural habitats as well as in organisms (Wilcox et al. 2018, MacLeod et al. 2021). Although plastic is robust, its integrity is constantly compromised by mechanical, physical, and chemical stressors, leading to fragmentation, abrasion. Thus, huge amounts of plastic particles are formed, including the microplastic (MP) size fraction of 1 µm to 5 mm. (Law and Thompson 2014, Meides et al. 2021, Mauel et al. 2022).

Plastic materials can cause injuries, entanglement or even suffocation, thus severely endangering wildlife (Marn et al. 2020). Oceanic plastic pollution dominated MP research, media focus, and the public discourse (Thompson et al. 2004, Lebreton et al. 2018), while terrestrial MP topics were underrepresented (He et al. 2020), despite the fact that soil acts as a sink for the majority of global MP (Horton et al. 2017).

Since 2010, scientists around the globe intensified their research of terrestrial MP, but with major focus on the northern hemisphere (He et al. 2020). Data concerning African MP and especially Sub-Saharan soil as depicted in previous studies is scarce with only 3.4% of MP datasets, making it a blind spot for this type of research (Jenkins et al. 2022). Notably, 92% of plastic waste is mismanaged in Kenya, underlining the assumption that littered plastic is omnipresent (IUCN-EA-QUANTIS 2020 et al. 2020). The environmental and health consequences of plastic pollution are largely unmonitored, leaving ecosystems and communities increasingly vulnerable to long-term contamination. Thus, it is a focus area in terms of plastic pollution that was already acknowledged in 2020 by the United Nations (IUCN-EA-QUANTIS 2020 et al. 2020). The African plastic pollution and its implications for humans poses considerable risks, as our societies are not only globalized, but our ecosystems are highly intertwined. An example of this linkage was the increasing number of “Saharan Dust Rain” events that gained attention in Europe (Varga et al. 2021). In a parallel fashion to these particles traveling hundreds and thousands of miles, MP might be transported over comparable routes as well (Petersen and Hubbart 2021). Hence, studying the effects of MP in Sub-Saharan soils not only helps to assess the risk potential for people in this particular region of the world, but rather for the global human civilization (Gomez et al. 2020, Jenkins et al. 2022). Thus, it is not surprising that Tchiechoua and Rillig (2025) recently highlighted the urgent need for MP research in Africa.

A multitude of plastic effects on terrestrial systems were reported including their function as vector for antibiotic resistances (Zhu et al. 2022), decrease of arthropod numbers such as mites and larvae in soil fauna experiments (Lin et al. 2020), alteration of hydrogen production upon MP exposure in woodlice guts (Lin et al. 2020, Hink et al. 2023), changes in the gut microbiome of earthworms (Holzinger et al. 2023, Hink et al. 2024), as well as agricultural soil properties (Souza Machado et al. 2019). Soil MP pollution deserves even more attention considering recent findings indicating plastic-driven enrichment of certain fungal human pathogens in the soil plastisphere (Gkoutselis et al. 2021, 2024). Globally, not only fungal pathogens were associated with plastic pollution, but also bacterial strains in aquatic (Kirstein et al. 2016) and in terrestrials systems (Rohrbach et al. 2022, Zhu et al. 2022). Furthermore, MP is considered to impact soil bacterial microbiomes and functionalities, e.g. by rearranging the prokaryotic community composition and by changing the genetic potentials for physiologically relevant processes (Sun et al. 2022a, Ng et al. 2021, Rüthi et al. 2023). So far it seems like there is no specific plastisphere microbiome, but rather a preferential attachment and enrichment on plastic surfaces also based on its properties (Li et al. 2022a, Cai et al. 2019, Bhagwat et al. 2021, Rohrbach et al. 2022). The ecological basis of the compositional alteration is a highly discussed topic and it became clear that the process is an interplay between stochastic or directed processes (Gundersen et al. 2021). Plastiphily and the expected deterministic community assembly that would follow is a vividly discussed neologism, but it needs further examination especially in open field studies (Sun et al. 2022b, Gkoutselis et al. 2024).

In addition, fungal and bacterial plastisphere communities are emerging research subjects, though archaea–MP interactions are often overlooked. The dominance of bacteria over archaea implies higher ecological relevance of the former, but the functional importance of archaea within terrestrial environments should not be underestimated (Offre et al. 2013). Interestingly, global archaeal biomass is estimated to contribute around 20% to prokaryotic biomass predominantly in oceanic habitats (Flemming and Wuertz 2019). In most studies from soil environments, they often represent only <10% relative abundance of the total prokaryotic community (Jaswal et al. 2019), but catalyse essential processes in biogeochemical cycles, such as ammonia oxidation and methanogenesis (Thauer et al. 2008, Flemming and Wuertz 2019, Jung et al. 2022). Ammonia-oxidizing archaea are more abundant than their bacterial counterparts in many soils (Nicol et al. 2008), and certain diazotrophic archaeal species can as well sequester N_2_ directly from the atmosphere during N-limitation (Dekas et al. 2009, Offre et al. 2013). Taking into account that plastic influx significantly alters biogeochemical processes catalysed by archaea, the interaction between the ubiquitous pollutant MP and the globally relevant archaea must be included into plastisphere studies (Gao et al. 2021a, Seeley et al. 2020, Rohrbach et al. 2022).

Evolution of efficient plastic biodegraders that will integrate the plastic carbon into the global C-cycle in the environment appears unlikely, but possible. Indeed, the biotechnological degradation of certain plastic and MP polymers is advancing at high rates (Lu et al. 2022, Sullivan et al. 2022). Despite these advances, it is unlikely that present and future MP or smaller fragments will entirely vanish from the Earth’s biosphere in the upcoming centuries, considering today’s knowledge (Lu et al. 2022, Sonke et al. 2022, Sullivan et al. 2022). The fact that certain MP types persist in most environments for multiple human generations necessitates the closing of knowledge gaps on how MPs interact with and alter the microbial community in Sub-Saharan soil ecosystems and on the African continent. Thus, we address the following hypotheses: (i) plastic-contamination has an effect on the abundance of archaea and shapes the composition of prokaryotic communities; (ii) plastisphere community assembly follows deterministic selection; (iii) plastisphere communities have distinct physiological genetic potentials, which differ from those of bulk soil communities; and (iv) the plastisphere contains a higher proportion of opportunistic pathogens compared to the bulk soil. Here, analyses of the archaeal and bacterial plastisphere communities in comparison with bulk soil originating from a Sub-Saharan area were performed and the occurrence of pathogens, plastic-degrading candidate taxa, degradative pathways, and the extent of copiotrophy were predicted based on genetic information. Furthermore, interaction networks were constructed and community assembly mechanisms determined.

## Materials and methods

### Sampling procedure and nucleic acid extraction

Plastisphere and bulk soil samples originated from a densely populated area, the surroundings of Siaya, Kenya at five different locations positioned as follows (Fig. S1): a landfill (S1; 0° 3′ 50.04″ N, 34° 16′ 54.479″ E), a roadside (S2; 0° 3′ 24.84″ N, 34° 18′ 19.8″ E), the Ramba marketplace (S3; 0° 3′ 24.84″ N, 34° 16′ 29.999″ E), a courtyard at Aringo Estate (S4; 0° 4′ 45.48″ N, 34° 16′ 48.359″ E), and another landfill in Siaya central (S5; 0° 3′ 49.32″ N, 34° 16′ 54.12″ E). The radius of the sampling area was 3 km (Fig. S1). Plastic debris and adjacent soil were collected from the different sampling sites and dry-stored prior to further processing in the laboratory as described in Gkoutselis et al. (2021). Five replicates were routinely analysed with the exception of S1 and S4, where one bulk soil and one MP sample, respectively, was lost during transport. Thus, four replicates of the bulk soil from S1 and the plastisphere from S4 were available, resulting in 24 soil and 24 plastic samples from five locations (i.e. 48 samples in total). Plastic debris were separated from bulk soil manually facilitated using light microscopy, and stored at –20°C prior to nucleic acid extraction. Nucleic acid extracts used in this study were the same as in Gkoutselis et al. (2021) and were prepared using the NucleoSpin^®^ Soil Kit (Machery Nagel, Düren, Germany) according to the manufacturer’s instructions.

### rRNA gene amplicon sequencing

For the prokaryotic community analysis, DNA extracts were processed according to the protocol provided by Illumina (San Diego, CA) as described in Rohrbach et al. (2022). The primers 515f(P) (Apprill et al. 2015) and 806R(A) (Parada et al. 2016) tagged with specific Illumina adapters were used to amplify archaeal and bacterial 16S rRNA genes as described previously (Rohrbach et al. 2022) and controlled using a 1.5% agarose gel. Purification of 16S rRNA gene amplicons was performed using MagSi-NGS^PREP^ Plus magnetic beads (Steinbrenner, Wiesenbach, Germany) following the manufacturer’s instructions. Sequencing libraries were prepared after indexing the amplicon polymerase chain reaction (PCR) products with Nextera XT v2 Primer Kit (Illumina) and sequenced on the iSeq-100 NGS instrument (Illumina). Sequencing data was demultiplexed using Illumina’s Local Run Manager. 16S rRNA gene sequences were analyzed using QIIME2 as described in Mlinar et al. (2020). Briefly, short reads (150 bp R1 reads) were quality filtered and denoised using the DADA2 pipeline, and taxonomic classification of amplicon sequence variants (ASVs) was performed using a classifier trained on 16S rRNA gene sequences (99% identity threshold, trimmed to the amplicon region excluding PCR primers) from the SILVA 138 database (www.arb-silva.com) with minor adjustments based on Oren (2021).

### Downstream analysis and data processing

Alpha-diversity of communities was examined using the plot_richness function based on the *phyloseq* package in R version 4.1.0 (McMurdie and Holmes 2013). Beta-diversity analyses were based on the Aitchison compositional distance metric available in the DEICODE plugin for QIIME2 (Martino et al. 2019). Taxonomic trait rankings and log-ratios of abundances between different sample groups were visualized using the R packages vegan 2.6–6.1, phyloseq 1.48.0, and tidyverse 2.0.0 (Jari Oksanen et al. 2009, McMurdie and Holmes 2013, Villanueva and Chen 2019, Wickham et al. 2019). Rarefaction curves were created using the R package vegan 2.6–6.1. Indicator analysis was conducted using the R package indicspecies 1.7.15 (Cáceres and Legendre 2009, Cáceres et al. 2011) with the multipatt function (r.g. parameter and 9999 permutations). Differential abundance analyses based on the negative binomial distribution was carried out using the R package DESeq2 1.44.0 with parametric fit type settings (Love et al. 2014). Venn diagram was generated applying the R package ggVennDiagramm 1.5.2 including taxa occurring in at least two samples (Gao et al. 2021b).

### *In silico* assignment of physiological traits and identification of potential pathogens

Metabolic activity potentials and traits of prokaryotic communities were acquired by database alignment of phylogenetic data (PICRUSt2; Douglas et al. 2020) implemented in QIIME2 and further processed using the MetaCyc database (Caspi et al. 2020). Functional annotation of prokaryotic taxa (FAPROTAX) as another tool for pathway abundance estimation was applied for comparison. This was performed by implementing a pipeline in QIIME according to the instructions from the Louca Lab (Louca et al. 2016). Further information regarding the catabolic potential of the tested compartments was addressed by calculating the rRNA operon (rrn) copy number. This was done using the ribosomal RNA operon copy number database (rrnDB; Stoddard et al. 2015). The rrn copy numbers were aligned with the lowest taxonomic level available and processed together with the abundance of each taxon for the respective sample. Mean values were then calculated according to the approach of Dai et al. (2022).


\begin{eqnarray*}
{rrn\ copy\ number} = \frac{{\mathop \sum \nolimits_{i = 1}^N {{S}_i}}}{{\mathop \sum \nolimits_{i = 1}^N \frac{{{{S}_i}}}{{{{n}_i}}}}},
\end{eqnarray*}

where *N* is the number of ASVs in a sample, *S_i_* is the sequence abundance of ASV*_i_*, and *n_i_* is the estimated *rrn copy number* of ASV*_i_*based on rrnDB.

We also aligned our data according to the microbial biodegradation of persistent organic pollutant database (mibPOPdb), giving an estimation for persistent organic pollutant degradation potentials (Li et al. 2024a, Ngara et al. 2022). The occurrence of pathogens in tested communities was examined by using the 16S rRNA gene based pathogen identification process (16SPIP) after aligning sequences with the database (Miao et al. 2017). Processing was done as previously reported (Rohrbach et al. 2022) In brief, database hits with a >99% similarity to pathogen reference sequences were identified as taxa of potential concern to human health. The relative abundance of these taxa in amplicon libraries was calculated based on the sequence data described in the previous section. *In vivo* pathogenicity of these identified taxa was not tested. A list of potential plastic-degrading organisms was acquired from the plastic biodegradation database (PlasticDB) provider website and compared with community data to identify putative plastic degrading organisms (Gambarini et al. 2022). Data were displayed using the pheatmap() and boxplot() functions in R.

### Network analyses

Correlation network analysis datasets were created using the SCNIC package in QIIME 2 and the sparCC function with a p-min-val of 0.7 and p-n-procs of 2 otherwise default settings were applied. The source codes on GitHub (https://github.com/lozuponelab/(q2-)/SCNIC) and sparCC were described earlier (Friedman and Alm 2012). Networks were created for the soil microbial community or the plastic-associated microbial community. Data were visualized and acquired using Gephi 0.10.1 software with the Fruchtermann–Reingold projection (Fruchterman and Reingold 1991).

### Neutral community model

Community assembly of a given habitat is driven by both stochastic (random dispersal) and deterministic (selection) factors (Nemergut et al. 2013, Gundersen et al. 2021). To evaluate the importance of these factors, we applied the neutral community model (NCM) established by Sloan et al. (2006) to determine community assembly mechanisms. When the relative frequency and the mean relative abundance of given ASVs correlates according to the null model, the assembly is solely driven by stochastically occurring events. Detailed calculations can be found in Gkoutselis et al. (2024). The analyses were conducted using a public R script (Burns et al. 2016). The NCM was applied separately to bulk soil and plastisphere as well as to the combined dataset. ASVs from each dataset were separated into three partitions depending on whether they occurred more frequently than (above-partition), less frequently than (below-partition) or as frequently as predicted by the NCM (neutral-partition) based on 95% confidence intervals. Ecologically, taxa above or below prediction are therefore considered as being actively selected for (selection) or against (exclusion) by the respective habitat, respectively (deterministic processes), while taxa within prediction are considered randomly assembled (neutral processes).

### Ecology of community assembly

Assembly processes can be further divided into five different processes, namely, homogeneous and heterogeneous selection, dispersal limitation, homogeneous dispersal, and drift. To calculate the contribution of each single factor, we applied the “infer community assembly mechanisms by phylogenetic bin-based null model analysis” (iCAMP) pipeline provided by GALAXY (https://usegalaxy.eu/) and created by Ning et al. (2020). The respective abundance table was acquired using QIIME2 as described above, and the phylogenetic tree was created using the QIIME phylogeny align to tree mafft fastree function and converted into a *.nwk file for further processing. The dataset from the iCAMP pipeline was divided into three compartments similar to the NCM and the mean values for each processes were displayed using R for plot generation according to Ceja-Navarro et al. (2021) and Sun et al. (2022b).

### Statistical analyses

Statistical evaluations were performed using SigmaPlot 13.0 (Systat Software Inc. San Jose, CA, USA) for tabular numeric data. If neither the initial model nor the model transformed with ln-, x^2^ or sqrt(x)-function met normality assumption, the Dunn’s *post hoc* test was applied after Kruskal–Wallis one way analysis of variance on ranks, while the others were analyzed using the parametric one-way ANOVA with Tukey’s *post hoc* test. Differences in community composition were statistically evaluated using the Bray–Curtis dissimilarity and the adonis2() function as applied by Seeley et al. (2020).

## Results

Plastisphere and soil samples derived from different sites in a Sub-Saharan region were utilized to examine *in situ* plastic impacts on microbial community structure and genetic potentials relevant to biogeochemical cycling. Filtering and denoising of reads derived from 48 samples resulted in a total of 2 483 097 reads with a median read count of 49 680 and a minimum of 37 902 reads per sample. Sequencing depth was sufficient for all samples as indicated by plateauing rarefaction curves (Fig. S2). 2912 ASVs were identified, of which 20 ASVs belonged to archaea, 2814 to bacteria, and the residual sequences were unassigned.

### Prokaryotic community composition differences

**Figure 1.**
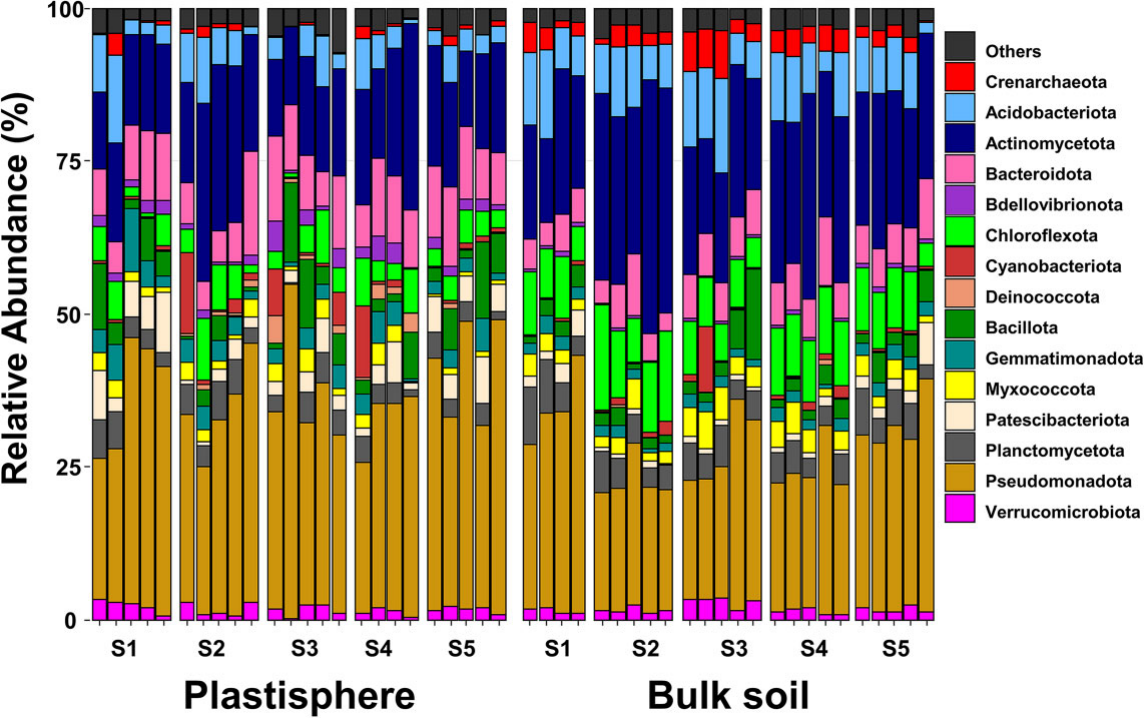
Plastisphere and bulk soil microbial community composition on phylum level. Relative abundances of taxa on phylum level were based on analyses of 16S rRNA gene amplicon sequences. Taxa with <2% relative abundance or unknown phyla in any of the samples were grouped as “others”. S1–S5 represent the sampling site (for details on sampling sites see experimental procedures).

Plastisphere and bulk soil communities were considered metacommunities (MC), if derived from different sites but same ecological compartment. On phylum level, both plastisphere and soil communities were dominated by Pseudomonadota and Actinomycetota accounting for roughly half of the total relative abundance, with a tendency toward higher relative abundances of Actinomycetota in soil MC (soil: 24 ± 1.3%; plastisphere: 18 ± 1%; *P* < .001) and Pseudomonadota in the plastisphere MC (plastisphere: 35 ± 1.5%; soil: 26 ± 1.3%; *P* < .001) (Fig. 1). Generally, less abundant taxa tended to be more heavily affected by the occurrence of plastic in comparison to more abundant taxa and groups (Table S1). The relative abundance of Deinococcota (*P* < .002), Bdellovibrionota (*P* < .001), and Hydrogenedentes (*P* < .116) was 3–5 times higher in the plastisphere relative to the bulk soil MC. Entotheonellaeota (*P* < .001), Elusimicrobiota (*P* < .001), and Methylomirabilota (*P* < .002), were 8–20 times more prominent in bulk soil than plastisphere MC. Chloroflexota (*P* < .001) and Bacillota (*P* < .248) accounted for up to 10% of total reads, and their relative abundances were 2 times higher in soil and 1.5 times lower in soil MC, respectively. Archaeal phyla, i.e. Crenarchaeota, appeared to be less abundant in the plastisphere than in the soil MC (*P* < .001).

**Figure 2.**
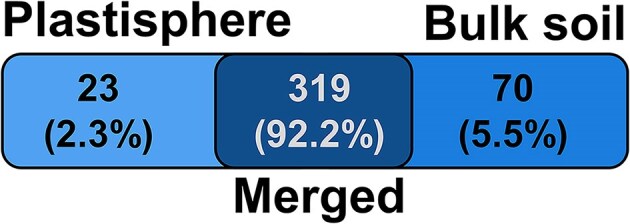
Venn diagram of plastic and bulk soil taxa. Numbers of common and specific taxa in the plastisphere and the bulk soil on family level. The color intensity correlates with the number of taxa and the mean relative abundances of the respective taxa in amplicon libraries are given in parentheses.

Analysis of specific and common taxa revealed that 319 taxa (92.2% mean relative abundance) were ubiquitous, while only 23 and 70 of taxa were plastic- and soil-specific, respectively (Fig. 2). Hence, specific taxa for both habitats were predominantly rare taxa, whereas abundant taxa were apparently generalists (Fig. 2). In addition, analyses of indicators were performed on family level. The most indicative families for plastisphere MC were Sphingomonadaceae, Bacteriovoracaceae, and Bdellovibrionaceae, whereas Gaiellaceae, Anaerolineae, and an Acidimicrobiia family were the most indicative taxa for bulk soil communities (Figs 1 and 2; Table S2).

### Prokaryotic alpha- and beta-diversity

**Figure 3.**
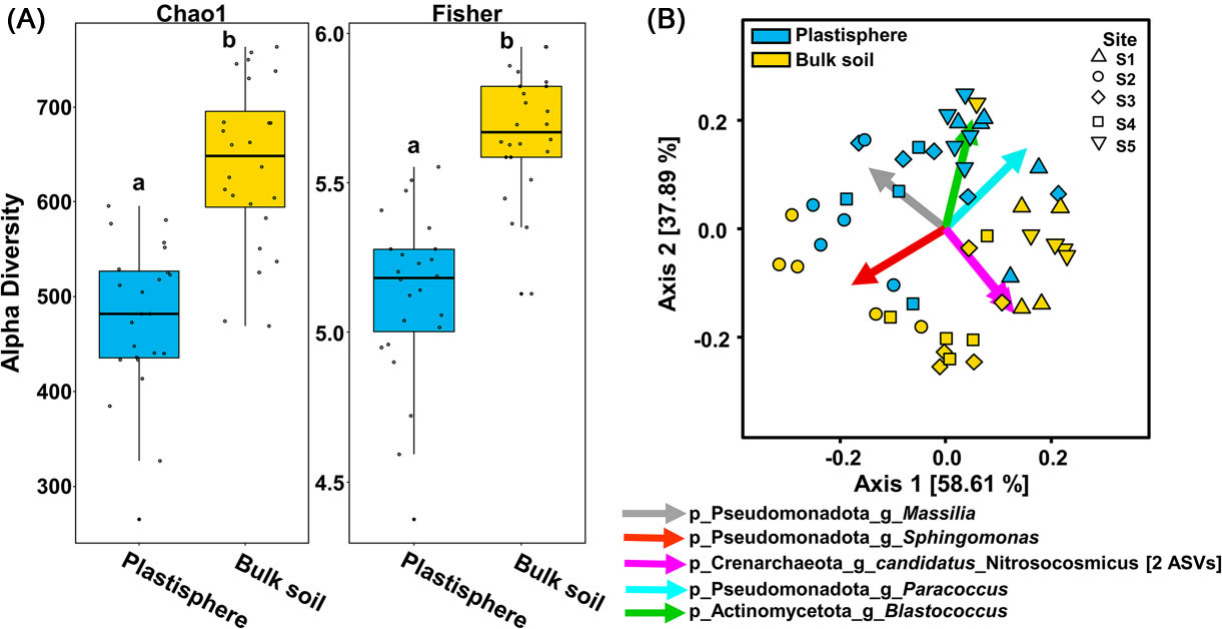
Properties of prokaryotic plastisphere and bulk soil communities. (A) Alpha-diversity measures based on Chao1 or Fisher indices. Lower-case letters indicate significant differences between MC (*P* < .05). (B) PCoA biplot based on robust Aitchison beta-diversity measures calculated for the total prokaryotic communities. Different shapes of symbols indicate different sampling sites: up-pointing triangle—S1 (landfill); circle—S2 (roadside); diamond—S3 (marketplace); square—S4 (courtyard); and down-pointing triangle—S5 (landfill). More details on sampling sites are presented in Fig. S1. Arrows indicate taxa that correlated best with the separation of the samples. The two magenta-colored arrows are assigned to “p_Crenarchaeota_g_*candidatus*_Nitrosocosmicus” and identify two indicative ASVs of this taxon. Lower-case letters indicate taxonomic rank as follows: p, phylum and g, genus.

**Figure 4.**
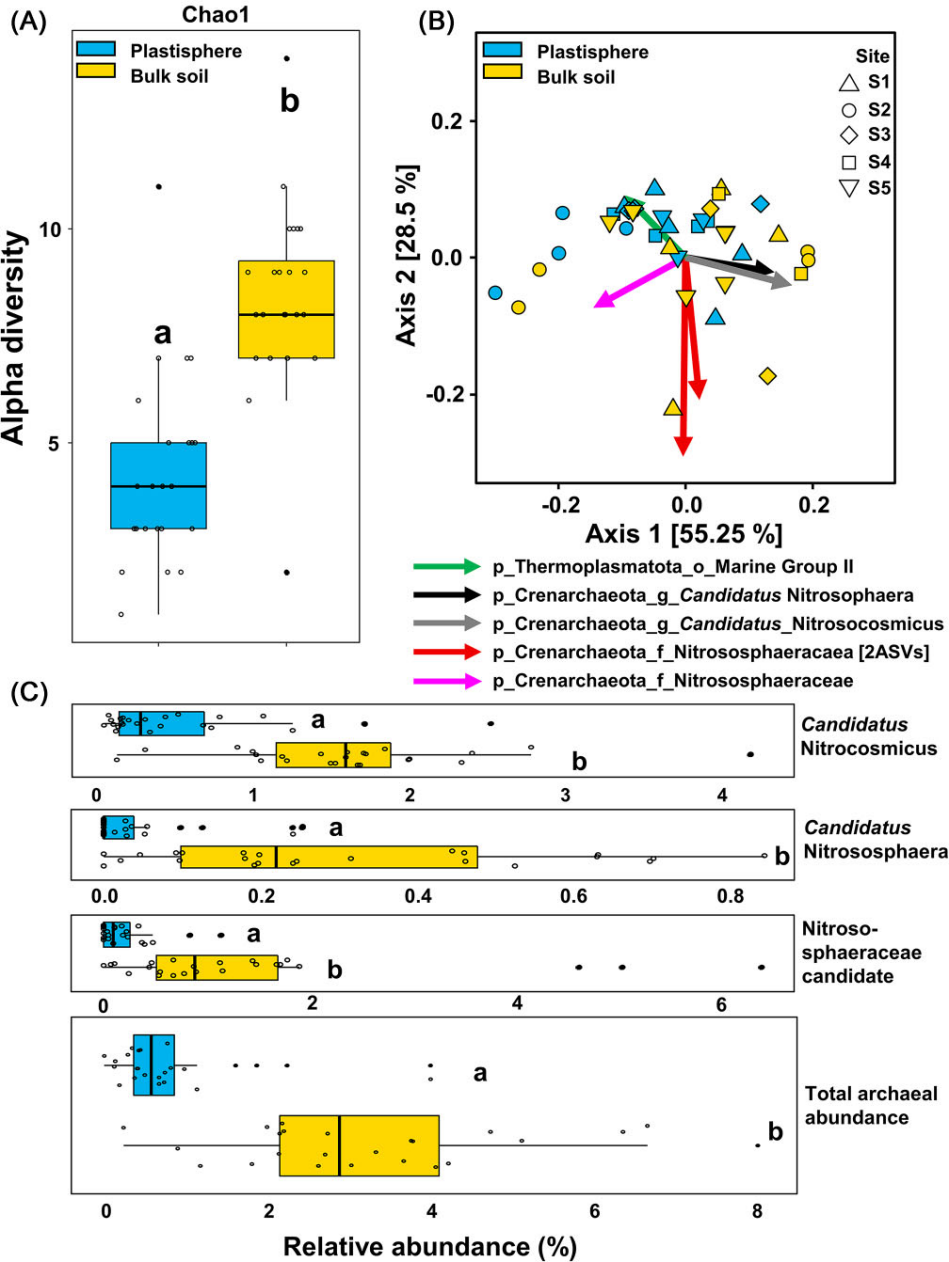
Properties of archaeal plastisphere and bulk soil communities. (A) Alpha-diversity measure for archaeal communities based on Chao1 indices. Lower-case letters indicate significant differences between MC (*P* < .05). (B) PCoA biplot based on robust Aitchison beta-diversity measures calculated for the archaeal community excluding unassigned taxa. Arrows represent Aitchison distance from the origin and indicate most important taxa correlated with the separation of the samples. Different shapes of symbols indicate different sampling sites: up-pointing triangle—S1 (landfill); circle—S2 (roadside); diamond—S3 (marketplace); square—S4 (courtyard); and down-pointing triangle—S5 (landfill). More details on sampling sites are presented in Fig. S1. The two red-colored arrows are assigned to “p_Crenarchaeota_f_Nitrososphaeracaea” and identify two indicative ASVs of this taxon. Lower-case letters indicate taxonomic rank as follows: p, phylum, f, family, and g, genus. (C) Boxplots displaying the relative abundance of ASVs in 16S rRNA gene amplicon libraries based on the total prokaryotic community of the most dominant archaea with lower-case letters indicating significant differences (*P* < .005).

The plastisphere samples were characterized by a significantly lower alpha-diversity in contrast to bulk soil communities (Fig. 3A and 4A). Beta-diversity measures based on Robust Aitchison distances indicated compositional differences between plastisphere and soil MC and explained over 96% of the total diversity (Fig. 3B). Permutational multivariate analysis of variance indicated significant differences between plastisphere and bulk soil communities (*P* < .001), whereas site-specific effects were marginal (*P* = .068). In line with the higher relative abundance of Nitrososphaeraceae in soil than in plastisphere communities (Fig. S4), *Candidatus* Nitrosocosmicus was one of the main drivers for beta-diversity (Fig. 3B). *Blastococcus, Massilia, Sphingomonas*, and *Paracoccus* were likewise important for the explanation of differences between plastisphere and bulk soil communities (Fig. 3B).

### Specific effect on the archaeal community

The high relative abundance of Crenarchaeota in amplicon libraries of the soil microbial community compared to their low relative abundance in the plastisphere was observed in most samples (Fig. 1 and Fig. S3). Consequently, the archaeal community was investigated in more detail. The strongest driver for the detectable differences was the abundance pattern of Nitrososphaeraceae with a mean relative abundance of around 3% in bulk soil communities in contrast to below 1% in plastisphere communities. Other archaeal families contributed to <0.5% to the total prokaryotic community in both bulk soil and plastisphere (Fig. S4). The assignment of Nitrososphaeraceae on a lower taxonomic level was limited, but both *Candidatus* Nitrosocosmicus and *Nitrososphaera* were identified, with the former being the most prominent genus within the Nitrososphaeraceae (Fig. S4 and Fig. 4C). The proportion of Nitrososphaeraceae in the total archaeal community did not differ significantly between plastic (93 ± 4.9%) and soil (99 ± 0.4%) compartments. Methanogenic archaea were rarely detectable (below 0.05% relative abundance) (Fig. S4). The alpha-diversity of archaea followed the same pattern as for the total prokaryotic communities, with a significant lower Chao index for plastisphere than for bulk soil communities (Fig. 4A). However, there was no significant difference in beta-diversity between archaeal soil and plastisphere MC (PERMANOVA: *P* > .05; Fig. 4B).

### Differences in the correlation network

**Figure 5.**
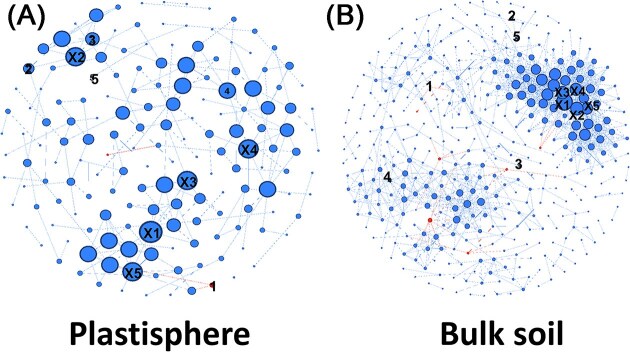
Correlation network of plastisphere and bulk soil communities. Community networks with taxa resembling nodes either for plastisphere sample or for pure bulk soil samples. The size of nodes corresponds to the number of connections (degree). Red nodes indicate archaeal taxa, blue nodes indicate bacterial taxa. The networks were created for plastisphere communities (A) and bulk soil communities (B). Numbers indicate the phylogenetic origin of respective nodes with greatest impact on beta diversity distance as follows: 1 *Candidatus* Nitrosocosmicus; 2 Pseudomonadota *Massilia*; 3 Pseudomonadota *Sphingomonas*; 4 Pseudomonadota *Paracoccocus*; and 5 Actinomycetota *Blastococcus*. Labels with X indicate the respective nodes with the highest degree of connectivity as follows: A X1 Chloroflexota_Ktedonobacteria_C0119; X2 Pseudomonadota *Belnapia*; X3 Acidobacteriota *Bryobacter*; X4 Pseudomonadota *Brevundimonas*; X5 Acidobacteriota Vicinamibacterales B X1 Actinomycetota *Acidothermus*; X2 Chloroflexota Ktedonobacterales; X3 Chloroflexota *Thermosporothrix*; X4 Pseudomonadota Burkholderia–Caballeronia–Paraburkholderia; and X5 Actinomycetota_*Jatrophihabitans*.

Community networks can provide useful information about the complexity of microbial communities. To address the question, whether archaea are not only repelled by plastic, but disturbed in their interactions with other taxa from a respective community, we calculated correlation networks. When checking for the connectivity, the network from soil was characterized by seven nodes of archaeal origin, whereas the plastic network contained just two nodes of archaeal origin (Fig. 5). The degree of connectivity, meaning the size and the number of edges of nodes, of archaea was higher on average in the bulk soil compared to the plastic network (Table 1 and Fig. 5). Soil MC network was characterized by a generally higher complexity, underlined by a higher number of nodes, edges, and average degree, whereas the plastisphere had a higher modularity (Fig. 5 and Table 1). Archaea were generally little connected within the networks, whereas *Paracoccus* was well-connected in the plastisphere network among the main drivers of beta-diversity. The most connected taxa were *Acidothermus* and *Ktedonobacteria* for bulk soil and plastisphere, respectively (Fig. 5 and Table 1).

**Table 1.** Network properties.

Network property	Plastisphere	Bulk soil
Number of nodes^a^	164	395
Number of edges^b^	203	1252
Modularity^c^	0.88	0.662
Network diameter^d^	7	9
Average path length^e^	2.038	2.862
Average degree^f^	1.238	3.17
Average clustering coefficient^g^	0.132	0.177

a Microbial taxon with at least one strong (SparCC <0.7) correlation.

b Number of connections/correlations obtained by SparCC anlysis.

c The capability of the nodes to form highly connected communities, that is, a structure with high density of between node connections (inferred by Gephi).

d The longest distance between nodes in the network, measured in number of edges (inferred by Gephi).

e Average network distance between all pair of nodes or the average length off all edges in the network (inferred by Gephi).

f The average number of connections per node in the network, that it, the node connectivity (inferred by Gephi).

g How nodes are embedded in their neighborhood and the degree to which they tend to cluster together (inferred by Gephi).

### Community assembly processes

**Figure 6.**
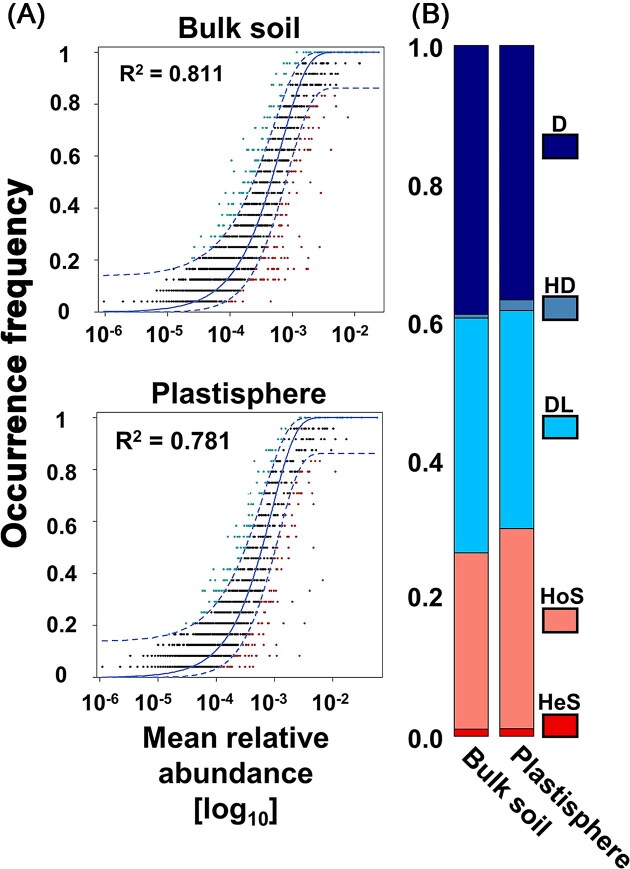
NCM analyses for determining the assembly mechanisms of prokaryotic communities in plastisphere and bulk soil. (A) Null model fit in which each point represents a prokaryotic ASV, and different colors indicate ASVs that occur more (cyan) or less frequently (dark red) than predicted by the neutral model. The solid blue lines resemble the optimum fit to the NCM and datapoints within the dashed blue lines are in the 95% confidence intervals of the model prediction. *R*^2^ indicates the fit quality of the neutral model. (B) Assembly processes within and between plastisphere and bulk soil communities based on inference of community assembly mechanisms by phylogenetic bin (iCAMP). Stochasticity-driven parameters are colored in blue and cyan, whereas the reddish colours represent the deterministic contributors. HeS—heterogeneous selection; HoS—homogeneous selection; DL—dispersal limitation; HD—homogeneous dispersal, and D—drift and others.

Community assembly of a given habitat is driven by a combination of stochastic (random dispersal) and deterministic (selection) factors (Nemergut et al. 2013, Gundersen et al. 2021). To evaluate the importance of these factors, we compared our data with null models. The correlation of tested communities for the plastic samples was 0.781, whereas those of bulk soil communities was 0.811 (Fig. 6A). This rather high correlation indicated that the community assembly was mainly driven by stochastic factors. The lower value for plastisphere indicated that plastics more strongly select for distinct prokaryotes in comparison to the bulk soil. Quantification of different community assembly processes underlined the dominance of stochastic events with a contribution of ~70% (Fig. 6B). Differences among process contributions were generally minor. iCAMP analysis revealed higher proportions of homogeneous selection and homogeneous dispersal for the plastisphere than soil MC, underlining a marginally higher selective pressure in the plastisphere (Fig. 6B).

### Physiology and potential pathogenicity of plastisphere and bulk soil communities

**Figure 7.**
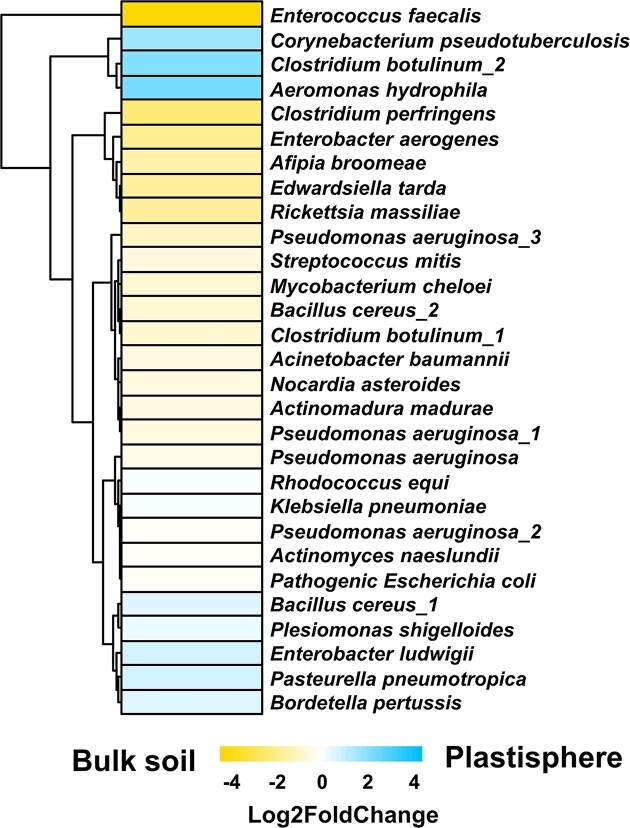
Estimation of pathogenic potential based on 16SPIP analysis. Differential abundance of putative bacterial pathogens between plastisphere and soil MC. The heat map represents log2fold changes (color coded) calculated by DEseq2. Positive and negative values indicate taxa that were enriched or depleted in the plastisphere relative to the bulk soil, respectively. Hierarchical clustering was applied on the abundance change patterns. Significant enrichments or depletion of disease-associated microbes were not detected.

Comparison of the obtained representative sequences with the 16SPIP database provided insights regarding the occurrence of potential human pathogens (Miao et al. 2017). Potential bacterial pathogens such as *Corynebacterium pseudotuberculosis, Clostridium botulinum*, and *Aeromonas hydrophila* were identified and had higher relative abundances in the plastisphere than bulk soil, although no significant enrichment was detected (Fig. 7). *Enterococcus faecalis* appeared to be depleted in the plastisphere.

**Figure 8.**
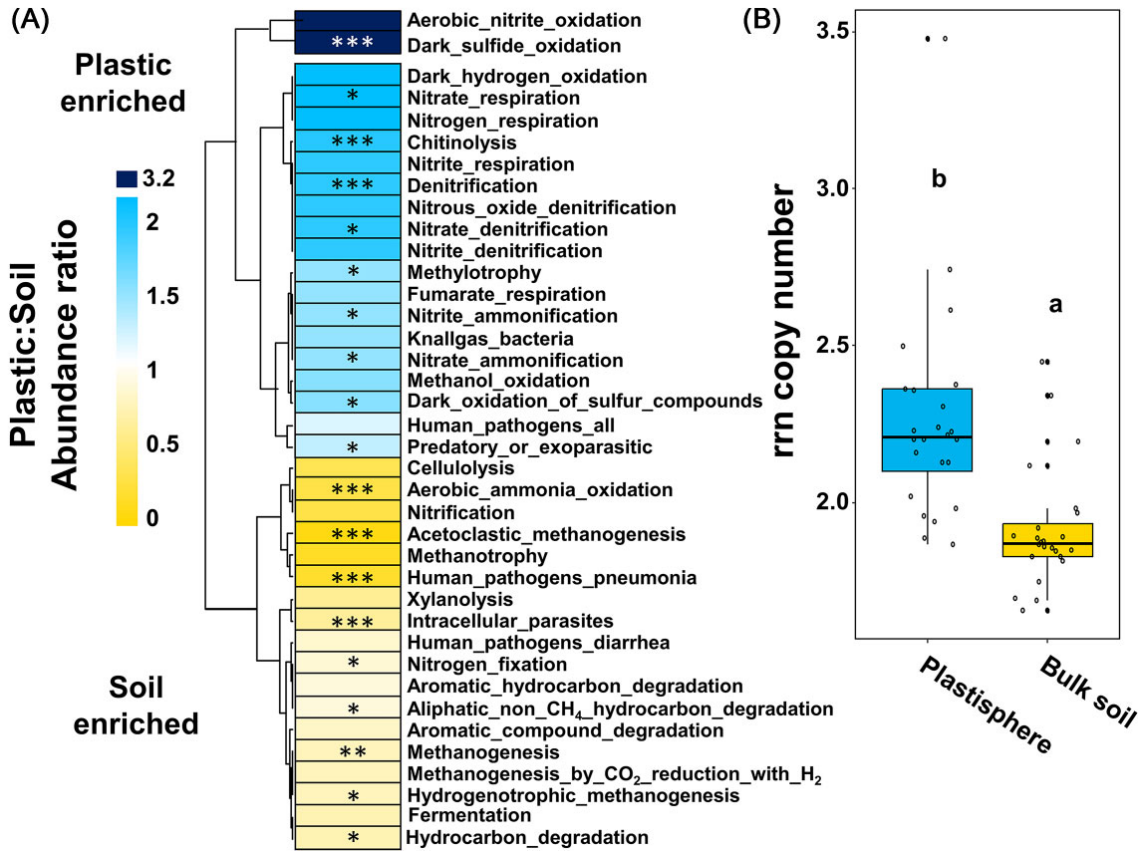
Physiological traits of tested plastisphere and bulk soil MC. Ratio of predicted pathway abundances of plastisphere and soil MC. Pathways which were more abundant in plastic than in soil samples are depicted in cyan. (A) Hierarchical clustering was applied to group those pathways, which follow a similar abundance pattern. *, **, and *** indicate significant differences between plastisphere and soil with a *P*-value of .05, .01, and .001, respectively. (B) rrn copy number in plastisphere and bulk soil communities. Lower-case letters indicate significant differences between rrn copy number occurrence between MC (*P* < .05).

PICRUSt2 was applied to examine, which xenobiotic degradation pathways were more abundant in either plastisphere or bulk soil communities (Douglas et al. 2020). Some pathways were enriched, when comparing soil and plastisphere samples. Nicotinate, creatinine, and taurine degradation pathways were more prominent in plastisphere than in bulk soil samples (Fig. S5). The opposite appeared for anaerobic aromatics and 4-coumarate degradation. FAPROTAX as prediction tool for more general pathways and biochemical processes indicated an increased genetic potential of predominantly denitrification related processes and chitinolytic traits in the plastisphere (Fig. 8A). Noteworthy are significantly increased ammonia-oxidizing potentials in soil, showing a depletion of ammonia oxidation potentials in the plastisphere relative to soil. In contrast to this, methane and aromatic degradation pathways in general tended to be less prevalent in the plastisphere community, which is in line with decreased compound degradation predictions acquired from PICRUSt2 predictions. To examine, whether the plastisphere MC is characterized by a specific degradation trait for recalcitrant xenobiotics, we investigated the genetic potential for persistent organic pollutant degradation. This analysis did not provide indications for an increased remediation potential in the plastisphere community (Fig. S6).

Lastly, we aimed to gain information regarding the occurrence of potential plastic-degrading microorganisms by aligning our data with known biodegraders from PlasticDB (Gambarini et al. 2022). Two ASVs were assigned to *Paracoccus denitrificans* and *Stenotrophomonas rhizopia*, which are capable of polyhydroxybutyrate and polyvinyl alcohol degradation, respectively. Both candidates were either depleted or not detectable in the plastisphere samples (Table S4). A comparison with known plastic degraders was performed on genus level, but no enrichment of these were detected for the plastisphere MC (Table S4 and Fig. S7). The rrn copy number estimator was generally low, and significantly increased in the plastisphere communities in comparison to bulk soil communities, indicating a better nutritional status of plastisphere than soil microbes, and a generally low nutritional level within the plastisphere and soil (Fig. 8B).

## Discussion

MP is a global pollutant and persists in all environments beyond human life spans (Persson et al. 2022). Plastic accumulation in terrestrial systems is more than a magnitude higher in comparison to marine systems (Horton et al. 2017). Considering that we live solely on landmasses, the reported strong MP impacts on terrestrial microbiomes potentially affecting N- and C-cycling, and the enrichment of putatively harmful microbes on MP in aquatic systems (Kirstein et al. 2016, Seeley et al. 2020, Rohrbach et al. 2022), it must be of fundamental interest to thoroughly understand plastic–microbe interactions in terrestrial ecosystems. Indeed, MP plays a role for human risk potentials due to vectoring of antibiotic resistances (Zhu et al. 2022) or pathogenic fungi (Gkoutselis et al. 2021, 2024)., We extend such insights in our study by assessing drivers of plastisphere community composition and consequences of MP for functional genetic potentials.

### Plastic shapes microbial community structure

Our results indicated that Actinomycetota and Pseudomonadota contributed to a large extent to the plastisphere community (Fig. 1). These findings are in line with other studies describing the plastisphere communities, even though additional other taxa are regularly described as dominating groups, namely Bacteroidota and Bacillota (Oberbeckmann et al. 2016, Rüthi et al. 2020, Zhu et al. 2022). Since such phyla occurred across all tested samples including soil samples in our study, they are important environmental groups naturally occurring in terrestrial and aquatic habitats (Sun et al. 2021). This supports the assumption that, although plastic surfaces select for distinct communities, they do not select for exclusive communities. This is in accordance with other reports (Vaksmaa et al. 2021b, Amaral-Zettler et al. 2020, Rohrbach et al. 2022), as a clear microbiome clustering between MP and surrounding environments was reported (Li et al. 2021, Sun et al. 2021, Rohrbach et al. 2022) (Fig. 3B). When checking for indicative bacterial taxa in the specific Sub-Saharan environment, the main drivers were part of the phyla Bdellovibrionota and Pseudomonadota (Table S2). Notably, the plastic-indicative families of Bdellovibrionota like Bacteriovoracaceae and Bdelovibrionaceae are characterized by a predatory life style feeding on Gram-negative bacteria including Pseudomonadota (Crossman et al. 2013), suggesting that predation is shaping community structure of biofilms in the plastisphere. The alpha diversity of the prokaryotic plastisphere metacommunity was significantly lower than that of the bulk soil (Fig. 3A). A similar pattern was observed in our previous study on mycobiomes from the same environment, suggesting a selective effect of the plastisphere environment on both, fungi and prokaryotes (Gkoutselis et al. 2021). In contrast to the strong divergence of plastisphere and bulk soil prokaryotic communities, the fungal beta-diversity measures suggested a higher degree of similarity of plastisphere and soil mycobiomes and thus a stronger impact of plastic surfaces on prokaryotes (Fig. 3B) (Gkoutselis et al. 2021).

Further analysis revealed 15 indicative families that tended to be rather selected by plastic (Table S2), which is in line with the cooccurrence network analysis. Plastic decreased the overall complexity of cooccurring networks in comparison to bulk soil communities, a finding that is supported by reports from terrestrial mesocosm incubations with polyethylene (PE) (Rong et al. 2021). Considering that lower complexity leads to a higher vulnerability against external, environmental stressors, terrestrial MP might have the potential to increase the risks of ecological disruption in nowadays rapidly changing ecosystems.

### Archaeal depletion in the plastisphere—a spotlight on functional guilds

Nitrososphaeraceae was the dominating archaeal family in tested samples. This group also contains the *Candidatus* Nitrosocosmicus, which is a reported ammonia oxidizer, growing optimally at high temperatures above 30°C, and thus might be relevant for the nitrogen cycle in Sub-Saharan soils (Lehtovirta-Morley et al. 2016). In general, we expected to find a large proportion of ammonia oxidizers, since the tested samples were acquired from top soil layers, which are constantly exposed to atmospheric oxygen levels (Stahl and La Torre 2012). Although archaea were generally depleted in the plastisphere, Crenarchaeaota was the dominant archaeal phylum on plastic debris from high seas, which is consistent with our data (Fig. 4C) (Woodall et al. 2018). Members of Nitrososphaeraceae were significantly depleted on tested plastic samples (Fig. S4), and indicative for soil samples (Table S1). As this matches the FAPROTAX-based metabolic potentials with significantly reduced ammonia-oxidizing potentials in plastisphere MCs, it supports partly the value of this database estimations (Fig. 8A). Since the proportion of Nitrososphaeraceae within all archaeal ASVs was not decreased, but rather the general abundance of all archaeal relative to bacterial families, we conclude that plastic has a general adverse effect on archaea (Figs 1 and 3C). Complementary metagenomic data were recently published, confirming a strong reduction of ammonium-oxidizing potentials within the plastisphere (Fortin et al. 2025). Reports on the plastisphere likewise indicate a depletion of archaea for other ecosystems (Vaksmaa et al. 2021b, Zhurina et al. 2022). Applying fluorescent *in situ* hybridization to marine samples revealed that such a depletion was particularly prominent for polyethylene (Vaksmaa et al. 2021b). Biofilm formation studies in bioreactors also revealed a decreased general archaeal attachment on polyethylene, whereas increased attachment rates were found on polyvinyl chloride and polycarbonate (Habouzit et al. 2011). As known from other studies, plastic types and properties, such as shore hardness are responsible for differential attachment and colonization patterns (Cai et al. 2019, Rohrbach et al. 2022). Tentatively, plastic chemical commodity induces the observed effects. Since the plastic debris samples were predominated by polyethylene material, the previous reports support our findings. It is widely accepted that bacterial and archaeal attachment and biofilm formation follow mostly the same pattern with some differences for instance in the pili and pili-like structures such as archaellins, fimbriae, or the hooked helical tubes called hami, which play a major role in biotic and abiotic attachment (van Wolferen et al. 2018). The molecular structure and resulting thermodynamic interaction of these structures with plastic surfaces could be the main driver of changed colonization patterns between bacteria and archaea. In contrast to plastiphilic fungi who are well adapted to hydrophobic attachment, archaea appear to be not suitable for plastic-attachment. A possible explanation might be the structural differences of the cell walls and membranes and possibly the dominant S-layer structure of archaea (Sleytr et al. 2014). Sleytr et al. (2014) reviewed the known literature regarding S-layer research and stated that archaea most often possess a hexagonal S-layer, which help to reduce adhesion of external molecules. Together with studies describing cell wall antibiotic repulsion, and S-layer-based antifouling properties of nano surfaces, this could add up to a rather hydrophilic than hydrophobic cell surface, which might result in a low adsorption capacity of archaea to plastics in the present study (Kandler and König 1998, Sleytr et al. 2014). However, underlying detailed mechanisms, such as possible S-layer-plastic surface interaction are interesting fields for further studies, which might open up new venues for surface-based biotechnologies. By combining these findings with the known physiology of detected taxa, it is expected that plastic pollution in aboveground environments will impair archaeal ammonia oxidation.

### Stochasticity drives microbiota communities

Null model assumption are regularly used to determine, whether community assembly is stochastic or deterministic (Gundersen et al. 2021). Indicator species analysis (Table S2) argues in favor of deterministic factors. Indeed, the formation of plastisphere communities in comparison to bulk soil communities was marginally more deterministically driven, although stochasticity was the major driver for bulk soil MC soil and plastisphere (Fig. 6). Generally, the fit was right in the range of another study from a terrestrial ecosystem (Li et al. 2024b), but much higher in comparison to marine studies (Sun et al. 2021). This might be based on the quantity of taxa with low relative abundance down to 10^–12^, whereas our model showed values only down to10^−6^ (Fig. 6). Li et al. (2022b) reported a much better fit of the NCM on the plastisphere community, when investigating the protistan community in soil environment. This might be supported by the occurrence of other support matrices like inorganic or organic surfaces such as wood, stone, or metal in terrestrial systems in contrast to unique plastic surfaces in marine systems. Sun et al. (2022b) reported a much lower proportion of stochastic effects in their mesocosm experiments relative to our study. Possible explanations might be the controlled conditions of a closed system, excluding a multitude of factors that are important in the field. A more detailed examination of community assembly mechanisms via iCAMP supported the prevalence of stochastic processes in our study. Community assembly was thus dominated by stochastic events, with a little higher importance of deterministic events in the plastisphere than in soil, which is supported by multiple previously reported studies (Li et al. 2022b, 2024b, Nemergut et al. 2013, Gundersen et al. 2021, Sun et al. 2021, 2024, Gkoutselis et al. 2024).

### Moderate effects on prokaryotic pathogens

MP is reported to play an alarming role in facilitating the distribution of antibiotic resistances (Zhu et al. 2022) as well as potential or opportunistic pathogens (Kirstein et al. 2016, Gkoutselis et al. 2021, 2024), and such investigations are compelling for the prokaryotic community in the Sub-Saharan soils of our study. Our study revealed only a trend toward higher numbers of taxa related to the pathogens *C. pseudotuberculosis, C. botulinum*, and *A. hydrophila* rather than a significant increase of pathogenic bacteria in the plastisphere (Fig. 7). This is further supported by the FAPROTAX estimations resulting in higher pathogenic potentials in bulk soil communities rather than in the plastisphere for pneumonia-associated pathogenicity potentials (Fig. 8A). Although *C. pseudotuberculosis* is primarily an animal pathogen, individual cases of infection with *C. pseudotuberculosis* have been reported, often leading to lymphadenopathy (Bregenzer et al. 1997, Dorella et al. 2006). *Clostridium botulinum* gained tragic fame through botulism, caused by strong neurotoxins (Lund and Peck 2013). Its growth prevention is one reason for high salt and nitrite contents in meat preservation (Lund and Peck 2013). *Aeromonas hydrophila* is known to cause infections of the human respiratory tract (Zhiyong et al. 2002). In contrast, previous findings revealed enrichment of fungal plastisphilic pathogens such as *Fusarium equiseti, Alternaria crassa*, and *Rhodoturula mucilaginosa* (Gkoutselis et al. 2021). This might be explained by different attachment processes of fungi, which can be promoted by hydrophobic hyphae and specialized enzymes (Tronchin et al. 2008). Nevertheless, the applied approach to identify prokaryotic pathogens (Miao et al. 2017) was entirely 16S rRNA gene sequence-based, i.e. identification of species that include pathogenic strains, without being able to discriminate between pathogenic and eventually nonpathogenic strains of the same species. For instance, bacterial-derived health issues are widely described for certain *E. coli* strains, possessing pathogenic or toxic factors on extrachromosomal elements like plasmids, albeit many other strains are nonpathogenic (Foxman 2010, Reid et al. 2022). Hence, we can refute our hypothesis of pathogen enrichment in the plastisphere in the investigated arid region and conclude from the present data that health risk potentials of terrestrial MPs in Sub-Saharan soils are more associated with fungi rather than bacteria as discussed previously (Gkoutselis et al. 2021, 2024).

### Impacts on metabolic potentials

MP exposure in different environments may not only alter soil properties (Rillig and Lehmann 2020), but may also affect metabolic processes (Seeley et al. 2020, Zhang et al. 2020, Rohrbach et al. 2022). In this study, PICRUSt2 and FAPROTAX predictions revealed notable increase of genetic nicotinate, creatinine, and taurine degradation potentials as well as denitrification, chitinolytic, and methylotrophic potentials within the plastisphere compared to the bulk soil community (Fig. 8 and Fig. S5). However, xenobiotic degradation pathways via the Nylon-6-oligomer pathway, involved in the degradation of polyamide (Vojdani and Giti 2015), were decreased in plastisphere communities (Fig. 8). Other studies reported a general stimulation of xenobiotic degradation within the plastisphere (Zhu et al. 2022). Since PICRUSt2 is using known genetic information, a lack of genomic data from the respective microorganisms might lead to an over- or underestimation of genetic-degradation potentials, even though the combination of different prediction approaches improves the interpretability of the acquired data. A chitinolytic trait indicate an adaption to attach to hydrophobic surfaces, and thus could support a plastiphilic lifestyle. However, genetic potentials lack information about actual activities as provided by transcription, protein, or process level data, but they provide first insights into the impact of plastics on genetic potentials of physiological traits that have evolved over longer periods of time.

Remarkably, Li et al. (2024b) reported that terrestrial plastisphere systems are characterized by a significant increase in their degradation potential for plastic and persistent organic pollutants, which could not be observed in our study and might be explained by the different climate zones in both studies. In this study, the rrn copy number was examined for both MC, indicating a significant higher number in the plastisphere samples. Generally, the observed values were low in comparison to other studies (Sun et al. 2022b, Dai et al. 2022). This indicates an environment with low levels of accessible metabolites. In contrast to these oligotrophic environments, nutrient-rich environments are often characterized by microbes with a high number of ribosomal RNA operons (Dai et al. 2022). Such organisms can process metabolites faster, gaining an advantage over organisms with a lower number of ribosomal RNA operons. Marginally higher values for the number of ribosomal RNA operons in the tested plastisphere community (Fig. 8) might indicate a release of additives or other nutrients from plastic debris, although the effects are far weaker in comparison to polylactic acid as a biologically more accessible polymer (Sun et al. 2022b), suggesting essentially an oligotrophic environment on Sub-Saharan plastic and the absence of or very limited microbial plastic polymer degradation.

The plastic degradation potential was assessed in more detail via comparison of assigned taxa with those provided by the publicly available database PlasticDB. Although well-known plastic-degrading taxa, such as *Ideonella sakaiensis* or *Aspergillus fumigatus* (Vaksmaa et al. 2021a, Yoshida et al. 2016, Gambarini et al. 2021, Zeghal et al. 2021) were not detected, multiple other plastic-degrading candidates on genus level and two on species level were found to be present in the analysed samples (Table S4). However, these taxa were not enriched within the plastisphere in comparison to bulk soil MC (Fig. S7). Tentatively, all reported plastic-degrading microorganisms are not solely growing on plastic, therefore the abundance of other, readily accessible, nutrients promoted their growth with a high likelihood. Nevertheless, PlasticDB analyses of genetic plastic-degradation potentials likewise suggest that bacterial plastic degradation was limited to nonexistent.

## Conclusion

The present study provides a significant extension of MP research in the Sub-Sahara and implications of MP pollution. In summary, new insights into how plastic fragments interact with the soil microbiota were acquired. The plastisphere community differs from the surrounding soil communities and represents an artificial microhabitat. We found strong indications for a repelling effect of common municipal plastic waste on archaea with possible effects on nitrogen cycling, which open a largely overlooked research subject, warranting further investigations focusing on molecular and process level. The strong impact of stochastic dispersal leads to the assumption that stochasticity is stronger in field studies in comparison to mesocosm incubations. Further, we examined the human health risk potential imposed by epiplastic prokaryotes and conclude that this was not high in the tested environment, despite mentioned limitations and reported health risks from other groups or organisms. Direct plastic-degradation potentials were low according to our data. This underlines the necessity of further studies, which more directly examine metabolic activities in the plastisphere. The more knowledge we can acquire regarding biodegradation, the more likely it is to develop efficient ways to limiting adverse effects from plastic pollution. Recent advances in biotechnological polyethylene, polyethylene terephthalate, and polystyrene recycling by harnessing prokaryotic potentials are promising (Tournier et al. 2020, Sullivan et al. 2022). This broad elucidation should help to pave the way to a research subject, which might open new facets of MP–microbe interactions.

## Supplementary Material

fiaf085_Supplemental_File

## Data Availability

Amplicon sequencing data have been deposited in the NCBI Sequence Read Archive (https://www.ncbi.nlm.nih.gov/sra) under the Bioproject number PRJNA1170214. All parameters used for each step in the QIIME2 workflow can be accessed via the provenance tab of the electronic data (Data repository DOI: 10.5281/zenodo.14747156). Further data are accessible upon reasonable request.
