# Supplementary material for: Microplastic impacts archaeal abundance, microbial communities, and their network connectivity in a Sub-Saharan soil environment
Source: FEMS Microbiol Ecol. 2025 Sep 2;101(10):fiaf085. doi: 10.1093/femsec/fiaf085 (PMC12481198; doi:10.1093/femsec/fiaf085)
Supplement: fiaf085_Supplemental_File [file fiaf085_supplemental_file.docx]

**Supplementary Information**

**Supplementary Tables**

**Table S1 Abundance distribution in bulk soil and plastisphere.**

| **Phylum** | **Relative abundance in plastisphere (%)*** | **Relative abundance in bulk soil abundance (%)*** | **P:S^#^** |
| --- | --- | --- | --- |
| **Deinococcota** | 0.82 | 0.16 | 5.21 |
| **Bdellovibrionota** | 1.76 | 0.49 | 3.61 |
| **Hydrogenedentes** | 0.02 | 0.01 | 3.19 |
| **Sumerlaeota** | 0.15 | 0.05 | 2.87 |
| **Abditibacteriota** | 0.22 | 0.08 | 2.8 |
| **Patescibacteriota** | 3.91 | 1.62 | 2.42 |
| **Cyanobacteriota** | 2.1 | 1.07 | 1.97 |
| **Gemmatimonadota** | 3.34 | 1.82 | 1.84 |
| **Bacillota** | 4.58 | 3.07 | 1.49 |
| **Bacteroidota** | 9.45 | 6.51 | 1.45 |
| **Nanoarchaeota** | 0.03 | 0.02 | 1.33 |
| **Pseudomonadota** | 35.03 | 26.29 | 1.33 |
| **Verrucomicrobiota** | 1.74 | 1.88 | 0.93 |
| **Armatimonadota** | 0.68 | 0.80 | 0.86 |
| **Fibrobacterota** | 0.03 | 0.04 | 0.83 |
| **Actinomycetota** | 17.85 | 24.06 | 0.74 |
| **Planctomycetota** | 3.62 | 5.05 | 0.72 |
| **Halobacterota** | 0.01 | 0.01 | 0.68 |
| **Myxococcota** | 1.81 | 3.16 | 0.57 |
| **Acidobacteriota** | 4.94 | 8.9 | 0.56 |
| **Chloroflexota** | 4.47 | 9.43 | 0.47 |
| **Desulfobacterota** | 0.02 | 0.04 | 0.47 |
| **Thermoplasmatota** | 0.01 | 0.01 | 0.41 |
| **Nitrospirota** | 0.10 | 0.32 | 0.32 |
| **Crenarchaeota** | 0.70 | 3.20 | 0.22 |
| **Elusimicrobiota** | 0 | 0.04 | 0.13 |
| **Methylomirabilota** | 0.01 | 0.10 | 0.1 |
| **Entotheonellaeota** | 0.01 | 0.14 | 0.05 |

* values represent the mean values of relative abundances of ASVs affiliated with a certain phylum and the total number of reads in 16S rRNA gene amplicon libraries.

^#^ ratio between the plastisphere and the bulk soil relative abundance as a proxy for plastic effects

**Table S2**

**Indicative families** **in plastisphere and bulk soil.**

| **Plastisphere** | | | **Bulk soil** | |
| --- | --- | --- | --- | --- |
| **Family*** | **Indval^#^** | | **Family*** | **Indval^#^** |
| Pseudomonadota _Sphingomonadaceae | 0.732 | | Actinomycetota _Gaiellaceae | 0.799 |
| Bdellovibrionota_Bacteriovoracaceae | 0.689 | | Chloroflexota_Anaerolineae_SBR1031 | 0.747 |
| Bdellovibrionota_Bdellovibrionaceae | 0.571 | | Actinomycetota _Acidimicrobiia_IMCC26256 | 0.734 |
| Abditibacteriota_Abditibacteriaceae | 0.570 | | Chloroflexota _KD4-96_ | 0.722 |
| Patescibacteriota_LWQ8 | 0.528 | | Actinomycetota _Solirubrobacterales_67-14 | 0.695 |
| Pseudomonadota _Comamonadaceae | 0.513 | | Actinomycetota _Micromonosporaceae | 0.665 |
| Bdellovibrionota_Oligoflexa [1ASV] | 0.509 | | Crenarchaeota_Nitrososphaeraceae | 0.664 |
| Bacteroidota_Hymenobacteraceae | 0.498 | | Actinomycetota _Ilumatobacteraceae | 0.648 |
| Pseudomonadota _Oxalobacteraceae | 0.496 | | Pseudomonadota_Gammaproteobacteria_uncult. [1ASV] | 0.644 |
| Bdellovibrionota_Oligoflexia_0319-6G20 | 0.481 | | Planctomycetota_Gemmataceae | 0.633 |
| Patescibacteriota_Saccharimonadales [1 [1ASV] | 0.478 | | Acidobacteriota_Thermoanaerobaculuaceae | 0.632 |
| Patescibacteriota_Saccharimonadales  [1ASV] | 0.470 | | Myxococcota_Polyangiales_BIrii41 | 0.628 |
| Cyanobacteriota_Sericytochromatia  [1 ASV] | 0.465 | | Myxococcota_bacteriap25 | 0.609 |
| Gemmatimonadota_Longimicrobiaceae | 0.441 | | Pseudomonadota Burkholderiales_TRA3-20 | 0.605 |
| Bacteroidota_Chitinophagaceae | 0.433 | | Acidobacteriota_Vicinamibacterales_uncult. [7 ASVs] | 0.602 |
|  |  |  | Acidobacteriota_Vicinamibacteraceae | 0.581 |
|  |  |  | Nitrospirota_Nitrospiraceae | 0.564 |
|  |  |  | Acidobacteriota_Bryobacteraceae | 0.547 |
|  |  |  | Chloroflexi_Roseiflexaceae | 0.542 |
|  |  |  | Pseudomonadota _Xanthobacteraceae | 0.540 |
|  |  |  | Actinomycetota _MB-A2-108 | 0.538 |
|  |  |  | Chloroflexota _Anaerolineae_SBR1031_A4b | 0.525 |
|  |  |  | Pseudomonadota _Steroidobacteraceae | 0.523 |
|  |  |  | Actinomycetota _Gaiellales_uncult, [10 ASVs] | 0.518 |
|  |  |  | Pseudomonadota _Nitrosomonadaceae | 0.511 |
|  |  |  | Actinomycetota _Solirubrobacteraceae | 0.508 |
|  |  |  | Pseudomonadota _Burkholderiales_SC-I-84 | 0.476 |
|  |  |  | Myxococcota_Sandaracinaceae | 0.472 |
|  |  |  | Actinomycetota _Microtrichales_uncult. [4 ASVs] | 0.463 |
|  |  |  | Chloroflexota _TK10 | 0.445 |
|  |  |  | Actinomycetota_Gaiellales [1ASV] | 0.443 |
|  |  |  | Chloroflexota _Gitt-GS-136 | 0.440 |
|  |  |  | Myxococcota_Haliangiaceae | 0.437 |
|  |  |  | Pseudomonadota_Elsterales_uncult.[3 ASVs] | 0.408 |

* taxa which contributed to the total relative abundance of at least 0.1% are indicated and number of ASVs given in parentheses in case of classification on family level was not applicable; uncult. - uncultured

^#^ only taxa with an Indval statistics of >0.4 and p value <0.005 were considered

**Table S3**

**Results of statistical Permutational Multivariate Analysis of Variance for archaeal community in plastisphere and bulk soil.**

| **Parameter** | **Sum of Squares** | **R^2^** | **F** | **p-value** |
| --- | --- | --- | --- | --- |
| **Location** | 0.1841 | 0.03498 | 1.6674 | 0.092 |
| **Treatment** | 0.9757 | 0.18541 | 10.47 | 0.001 |

**Table S4**

**PlasticDB annotated plastic degrading prokaryotes in plastisphere and bulk soil.**

Mean relative abundances with standard deviations of annotated putative plastic biodegraders are displayed. Ratio P:S resembles the abundance ratio between plastic and soil communities. Genus and Species are marked, when the candidate was annotated at the respective taxonomic level.

| **Plastic biodegrader** | **Genus** | **Species** | **Plastisphere** | **Bulk soil** | **Ratio P:S** |  |
| --- | --- | --- | --- | --- | --- | --- |
|  |  |  |  |  |  |  |
| ***Brevundimonas*** | | X |  | 0,006 ± 0.028 | 0.000 ± 0 | nA |
| ***Sphingomonas laterariae*** | | X |  | 0.005 ± 0.018 | 0.000 ± 0 | nA |
| ***Sphingomonas oligophenolica*** | | X |  | 0.003 ± 0.012 | 0.000 ± 0 | nA |
| ***Sphingomonas rhizophila*** | | X |  | 0.002 ± 0.008 | 0.000 ± 0 | nA |
| **uncultured endolithic** | | X |  | 0.003 ± 0.012 | 0.000 ± 0 | nA |
| **Janthinobacterium** | | X |  | 0.002 ± 0.009 | 0.000 ± 0 | nA |
| **Massilia** | | X |  | 0.002 ± 0.011 | 0.000 ± 0 | nA |
| ***Halomonas*** | | X |  | 0.003 ± 0.013 | 0.000 ± 0 | nA |
| ***Brevundimonas basaltis*** | | X |  | 0.065 ± 0.304 | 0.008 ± 0.015 | 8.42 |
| ***Brevundimonas terrae*** | | X |  | 0.002 ± 0.012 | 0.001 ± 0.003 | 4.65 |
| ***Bacillus graminis*** | | X |  | 0.001 ± 0.005 | 0.000 ± 0.002 | 3.26 |
| ***Sphingomonas agri*** | | X |  | 0.011 ± 0.029 | 0.004 ± 0.008 | 2.81 |
| ***Sphingomonas*** | | X |  | 0.101 ± 0.15 | 0.041 ± 0.06 | 2.45 |
| ***Sphingomonas formosensis*** | | X |  | 0.174 ± 0.313 | 0.072 ± 0.09 | 2.43 |
| ***Sphingomonas kaistensis*** | | X |  | 0.008 ± 0.038 | 0.004 ± 0.018 | 2.12 |
| **uncultured Sphingomonadaceae** | | X |  | 0.013 ± 0.049 | 0.006 ± 0.022 | 2.09 |
| ***Streptococcus*** | | X |  | 0.11 ± 0.191 | 0.053 ± 0.087 | 2.08 |
| **Bacterium KR** | | X |  | 3.114 ± 1.593 | 1.515 ± 0.614 | 2.06 |
| **uncultured Caulobacteraceae** | | X |  | 0.002 ± 0.009 | 0.001 ± 0.005 | 2.03 |
| ***Anoxybacillus toebii*** | | X |  | 0.024 ± 0.047 | 0.012 ± 0.021 | 2.01 |
| **Bacterium C0297-B0199** | | X |  | 0.016 ± 0.042 | 0.008 ± 0.024 | 1.88 |
| ***Sphingomonas oryziterrae*** | | X |  | 0.08 ± 0.09 | 0.043 ± 0.057 | 1.87 |
| ***Brevundimonas alba*** | | X |  | 0.001 ± 0.006 | 0.001 ± 0.003 | 1.67 |
| ***Xanthomonas*** | | X |  | 0.007 ± 0.029 | 0.004 ± 0.018 | 1.67 |
| ***Acinetobacter indicus*** | | X |  | 0.001 ± 0.002 | 0.000 ± 0.002 | 1.57 |
| ***Sphingomonas jaspsi*** | | X |  | 0.287 ± 0.273 | 0.231 ± 0.149 | 1.25 |
| ***Bacillus trypoxylicola*** | | X |  | 0.043 ± 0.061 | 0.035 ± 0.074 | 1.2 |
| ***Sphingomonas metalli*** | | X |  | 0.015 ± 0.029 | 0.013 ± 0.023 | 1.13 |
| ***Sphingomonas zeicaulis*** | | X |  | 0.018 ± 0.032 | 0.017 ± 0.024 | 1.1 |
| ***Sphingomonas cynarae*** | | X |  | 0.012 ± 0.036 | 0.011 ± 0.032 | 1.02 |
| ***Sphingomonas koreensis*** | | X |  | 0.013 ± 0.035 | 0.013 ± 0.024 | 1.01 |
| ***Cupriavidus pampae*** | | X |  | 0.018 ± 0.029 | 0.018 ± 0.028 | 1 |
| ***Cupriavidus*** | | X |  | 0.004 ± 0.016 | 0.005 ± 0.016 | 0.87 |
| ***Geobacillus*** | | X |  | 0.034 ± 0.105 | 0.043 ± 0.077 | 0.79 |
| ***Sphingomonas asaccharolytica*** | | X |  | 0.004 ± 0.021 | 0.006 ± 0.022 | 0.68 |
| ***Pseudomonas jessenii*** | | X |  | 0.005 ± 0.02 | 0.008 ± 0.027 | 0.65 |
| ***Sphingomonas guangdongensis*** | | X |  | 0.022 ± 0.039 | 0.035 ± 0.045 | 0.61 |
| ***Sphingomonas flava*** | | X |  | 0.001 ± 0.004 | 0.001 ± 0.007 | 0.59 |
| **uncultured Clostridiales** | | X |  | 0.016 ± 0.039 | 0.028 ± 0.063 | 0.59 |
| ***Thermobacillus* sp.** | | X |  | 0.066 ± 0.086 | 0.123 ± 0.087 | 0.53 |
| ***Massilia albidiflava*** | | X |  | 0.1 ± 0.149 | 0.203 ± 0.165 | 0.49 |
| ***Paracoccus kocurii*** | | X |  | 0.113 ± 0.099 | 0.253 ± 0.189 | 0.45 |
| ***Bacillus funiculus*** | | X |  | 0.006 ± 0.021 | 0.018 ± 0.027 | 0.33 |
| **uncultured bacterium** | | X |  | 0.007 ± 0.028 | 0.023 ± 0.043 | 0.28 |
| ***Gracilibacillus*** | | X |  | 0.002 ± 0.006 | 0.007 ± 0.023 | 0.28 |
| ***Melaminivora sp.*** | | X |  | 0.016 ± 0.017 | 0.085 ± 0.401 | 0.19 |
| ***Pseudoxanthomonas*** | | X |  | 0.001 ± 0.007 | 0.007 ± 0.035 | 0.19 |
| ***Bacillus clausii*** | | X |  | 0.001 ± 0.003 | 0.004 ± 0.014 | 0.16 |
| ***Sphingomonas daechungensis*** | | X |  | 0.003 ± 0.012 | 0.027 ± 0.046 | 0.09 |
| ***Bacillus*** | | X |  | 0.001 ± 0.004 | 0.021 ± 0.041 | 0.04 |
| ***Paracoccus denitrificans*** | | X | X | 0.001 ± 0.004 | 0.043 ± 0.055 | 0.02 |
| ***Stenotrophomonas rhizophila*** | | X | X | 0.000 ± 0 | 0.001 ± 0.005 | nA |
| ***Bacillus infernus*** | | X |  | 0.000 ± 0 | 0.003 ± 0.014 | nA |
| ***Bacillus thermolactis*** | | X |  | 0.000 ± 0 | 0.001 ± 0.004 | nA |
| ***Paracoccus*** | | X |  | 0.000 ± 0 | 0.001 ± 0.003 | nA |
| ***Sphingomonas difficilis*** | | X |  | 0.000 ± 0 | 0.003 ± 0.009 | nA |
| ***Sphingomonas ginsengisoli*** | | X |  | 0.000 ± 0 | 0.002 ± 0.007 | nA |
| ***Sphingomonas hengshuiensis*** | | X |  | 0.000 ± 0 | 0.001 ± 0.005 | nA |
| ***Sphingomonas soli*** | | X |  | 0.000 ± 0 | 0.002 ± 0.011 | nA |
| ***Sphingopyxis*** | | X |  | 0.000 ± 0 | 0.003 ± 0.015 | nA |
| ***Ralstonia*** | | X |  | 0.000 ± 0 | 0.006 ± 0.017 | nA |
| ***Duganella*** | | X |  | 0.000 ± 0 | 0.007 ± 0.013 | nA |
| ***Undibacterium*** | | X |  | 0.000 ± 0 | 0.003 ± 0.016 | nA |
| ***Acinetobacter*** | | X |  | 0.000 ± 0 | 0.003 ± 0.007 | nA |
| ***Ssychrobacter meningitidis*** | | X |  | 0.000 ± 0 | 0.046 ± 0.028 | nA |
| ***Pseudomonas*** | | X |  | 0.000 ± 0 | 0.009 ± 0.039 | nA |
| ***Sseudomonas resinovorans*** | | X |  | 0.000 ± 0 | 0.001 ± 0.004 | nA |
| ***Actinobacterium* A15** | | X |  | 0.000 ± 0 | 0.004 ± 0.019 | nA |
| ***Stenotrophomonas*** | | X |  | 0.000 ± 0 | 0.001 ± 0.005 | nA |

**Supplementary Figures**

**
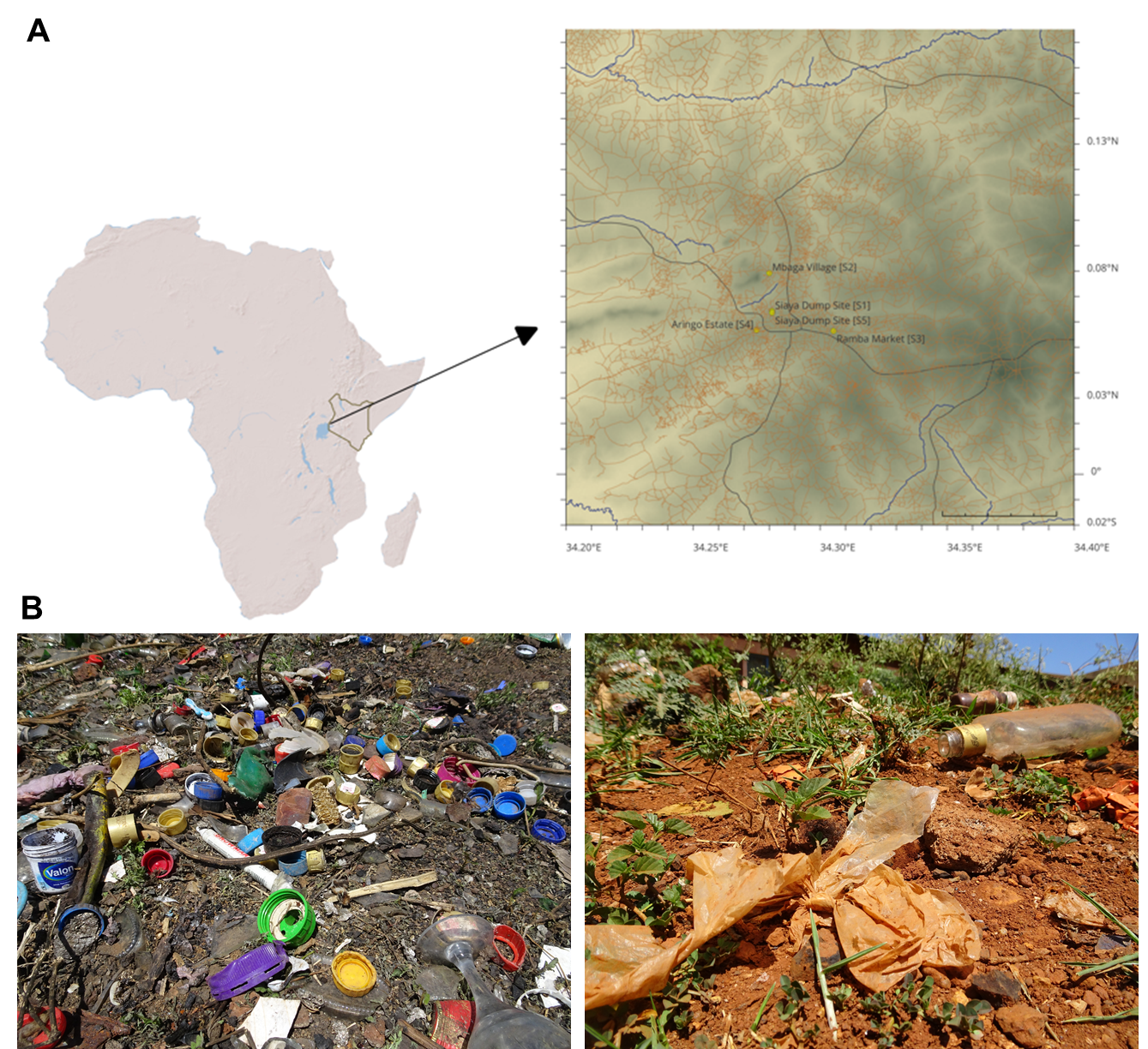
**

**Figure S1. Overview of sampling sites.** Geographical location of the sampling sites S1-S5 in the municipal area of Siaya, Kenya (A). In black are highways or tarmac roads, whereas brown indicates unpaved murrams and blue indicates water surfaces. The scale bar represents 5 km. Representative pictures of the Siaya municipal area to illustrate the degree of pollution at the sampling sites (B).

**
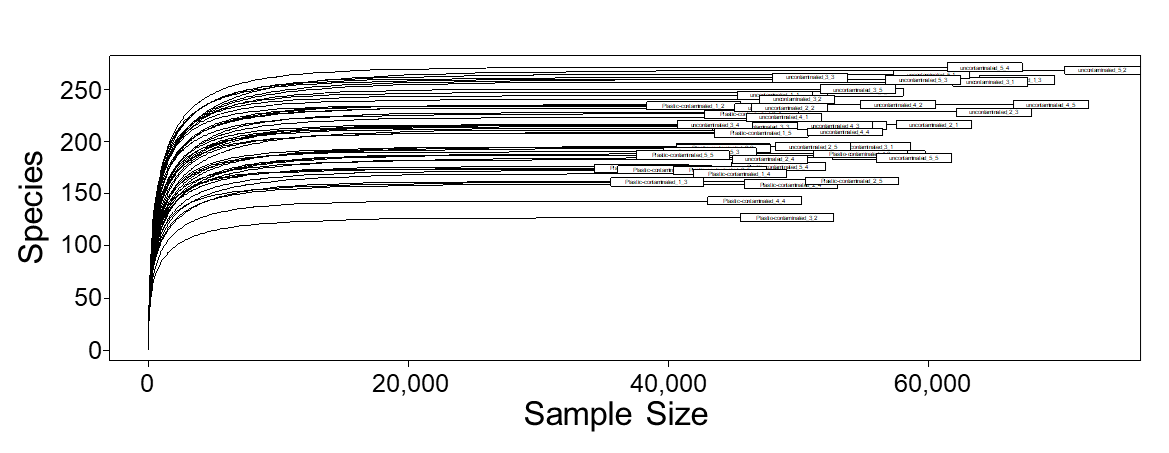
**

**Figure S2. Rarefaction curves of sequencing results.**


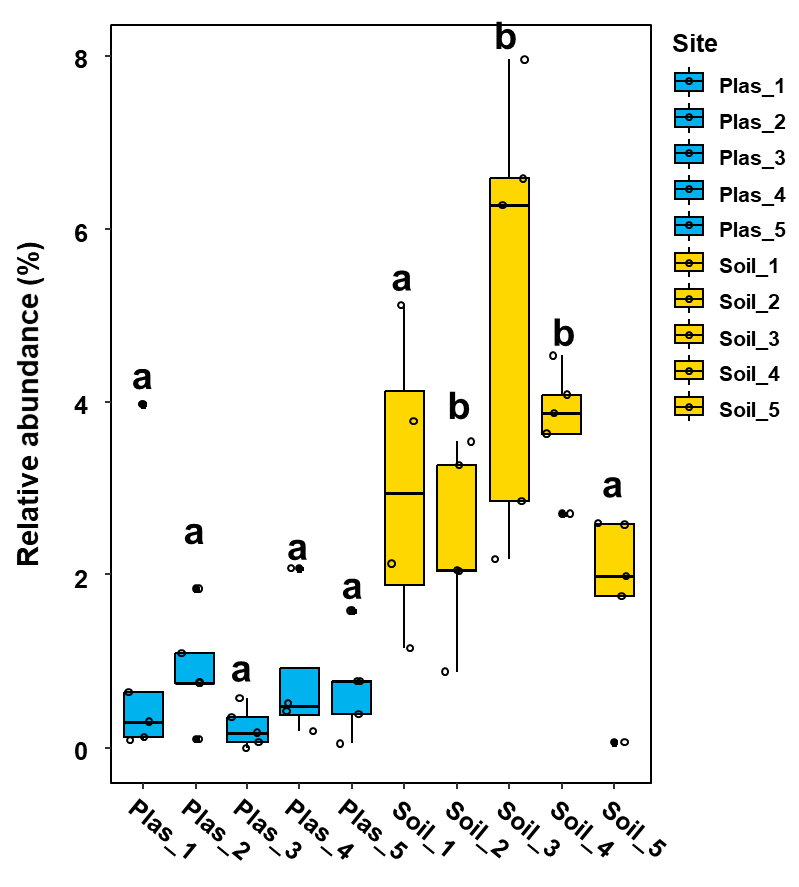


**Figure S3. Relative abundance of archaea-assigned reads among the different environmental habitats.** Plas – Plastisphere samples as subsamples from the total plastisphere MC; Soil – Bulk soil samples as subsamples from the total soil MC. Numbers in the sample names indicate the sampling site from S1-S5. Lower-case letters indicate significant differences (p< 0.05) between plastisphere and bulk soil samples from a specific sample site. Statistical analyses were performed as described in the material and method section in the main text.


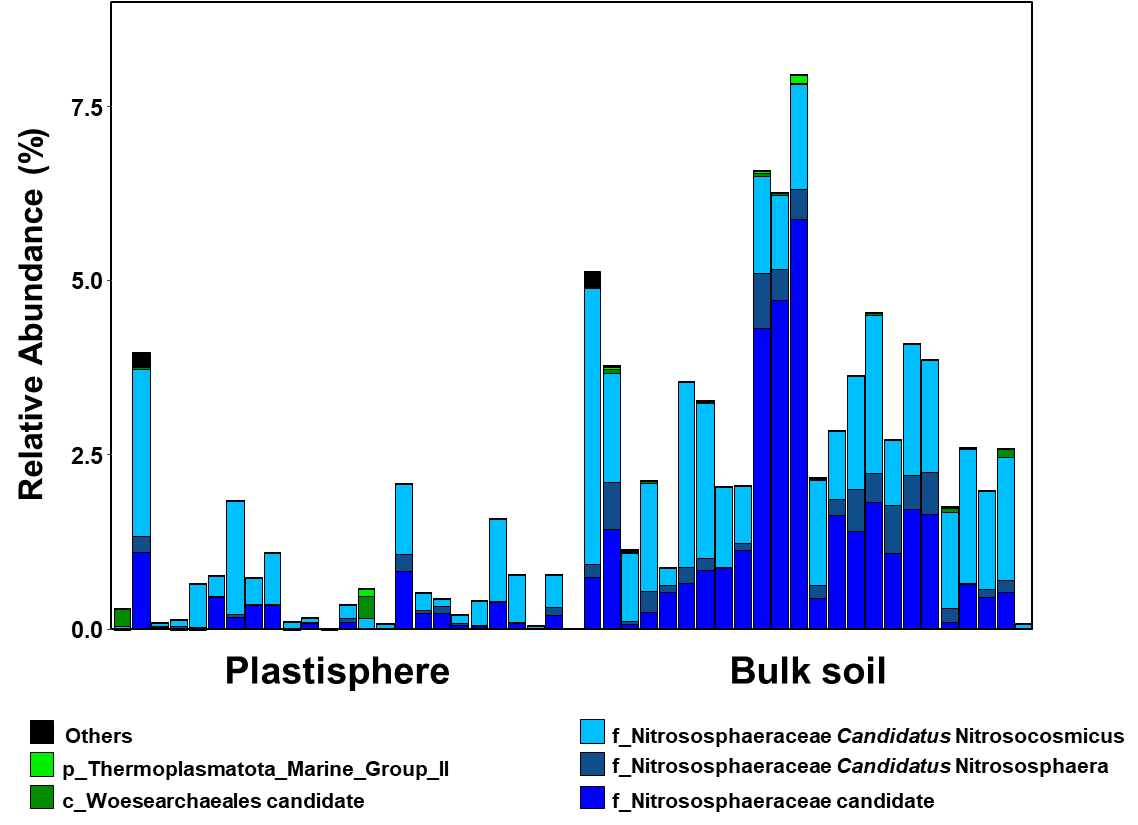


**Figure S4. Relative abundance of archaeal families in the plastisphere and bulk soil.** Relative abundances of archaeal ASVs in 16S rRNA gene amplicon libraries are displayed. “Others” include rarely detected taxa like *Methanosarcina* and *Methanobacterium* (on average < 0.01 % relative abundance). p, c and f indicate phylum, class and family taxa, respectively.


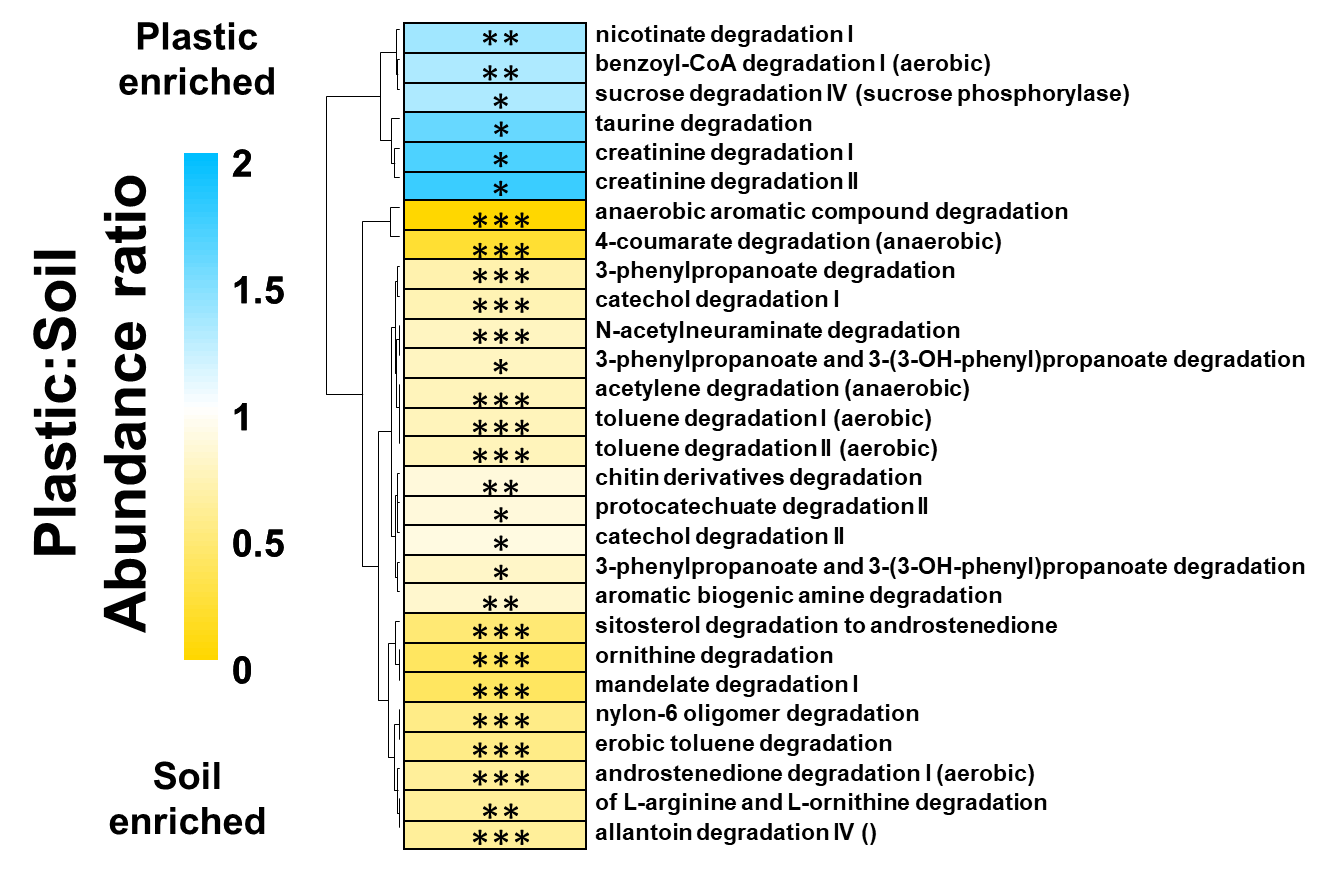


**Figure S5. Physiological traits of tested metacommunities.** Ratios of predicted pathway abundances of plastisphere and soil metacommunities based on PICRUSt2 analysis. Pathways which were more abundant in plastic samples are depicted in cyan, and pathways that were more abundant in soil are depicted in gold. Hierarchical clustering was applied to group those pathways which follow a comparable abundance pattern. *, ** and *** indicate significant differences between plastisphere and bulk soil with a p value of 0.05, 0.01 and 0.001, respectively**.**

**
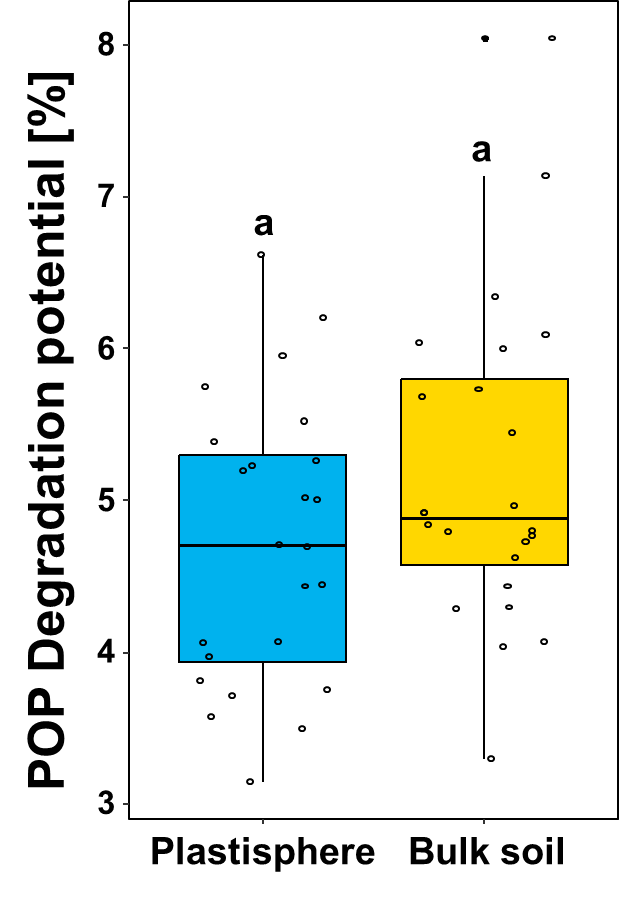
**

**Figure S6. Persistent organic pollutant degradation potentials in the plastisphere and bulk soil.** Relative abundance of taxa on family level associated with persistent organic pollutant degradation in plastisphere and bulk soil communities based on MibPOPdb analysis are displayed. Lower-case letters indicate significant differences (p< 0.05).

**
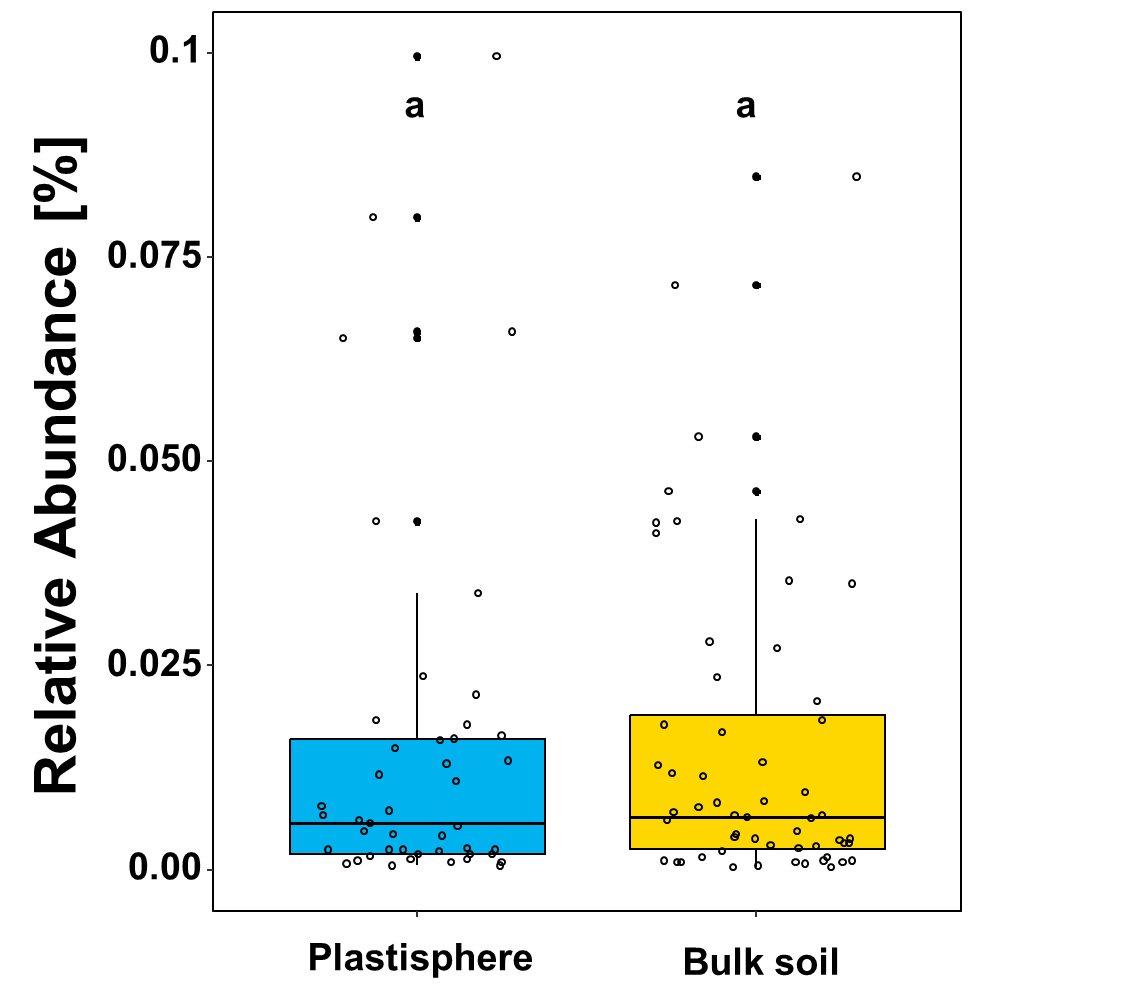
**

**Figure S7. Boxplots of relative abundance of annotated plastic degrading organisms in plastisphere and bulk soil.** Relative abundance of plastic biodegradation-associated candidates is displayed. Based on p-value < 0.05, no significant differences were detectable as indicated by lower-case letters
